# Uncovering periodic patterns of space use in animal tracking data with periodograms, including a new algorithm for the Lomb-Scargle periodogram and improved randomization tests

**DOI:** 10.1186/s40462-016-0084-7

**Published:** 2016-08-01

**Authors:** Guillaume Péron, Chris H. Fleming, Rogerio C. de Paula, Justin M. Calabrese

**Affiliations:** 1 Smithsonian Conservation Biology Institute, National Zoological Park, Front Royal, VA 22630 USA; 2 Department of Biology, University of Maryland, College Park, MD 20742 USA; 3 National Research Center for Carnivore Conservation (CENAP/ICMBio), Atibaia, Sao Paulo Brazil

**Keywords:** Periodicity, Circadian, Central place foraging, Autocorrelation, Activity cycles, Behavior

## Abstract

**Background:**

Periodicity in activity level (rest/activity cycles) is ubiquitous in nature, but whether and how these periodicities translate into periodic patterns of space use by animals is much less documented. Here we introduce an analytical protocol based on the Lomb-Scargle periodogram (LSP) to facilitate exploration of animal tracking datasets for periodic patterns. The LSP accommodates missing observations and variation in the sampling intervals of the location time series.

**Results:**

We describe a new, fast algorithm to compute the LSP. The gain in speed compared to other R implementations of the LSP makes it tractable to analyze long datasets (>10^6^ records). We also give a detailed primer on periodicity analysis, focusing on the specificities of movement data. In particular, we warn against the risk of flawed inference when the sampling schedule creates artefactual periodicities and we introduce a new statistical test of periodicity that accommodates temporally autocorrelated background noise. Applying our LSP-based analytical protocol to tracking data from three species revealed that an ungulate exhibited periodicity in its movement speed but not in its locations, that a central place-foraging seabird tracked moon phase, and that the movements of a range-resident canid included a daily patrolling component that was initially masked by the stochasticity of the movements.

**Conclusion:**

The new, fast algorithm tailored for movement data analysis and now available in the R-package ctmm makes the LSP a convenient exploratory tool to detect periodic patterns in animal movement data.

**Electronic supplementary material:**

The online version of this article (doi:10.1186/s40462-016-0084-7) contains supplementary material, which is available to authorized users.

## Background

Periodic patterns of space use, such as daily routines [1] and annual migrations [2], are a particular form of temporal autocorrelation in animal tracking data that has been largely ignored so far by movement modeling efforts. Periodic behaviors nevertheless constitute manifestations of complex, finely-tuned suites of behaviors that have been shaped by natural patterns of spatiotemporal variation in resources and risk [3, 4], so that their occurrence, or their absence, is often central to the ecology of species and individuals [5]. In particular, animals’ responses to lunar cycles [6] and resource depletion/recovery cycles [7] remain poorly described in most cases, and much insight could potentially be gained from analyzing animal movements with respect to those cycles. Memory effects, cognitive abilities [8, 9], and social interactions [10] represent other intriguing pathways through which periodicities could emerge in animal movement. In addition, periodic patterns of space use represent a major violation of the assumptions of commonly used stochastic movement models [11, 12]. Consequently, neglecting periodic behaviors when they occur can bias analyses that condition on fitted movement models, including home range size estimation and resource utilization.

The phrase “periodic pattern of space use” is, however, not to be confused with “periodicity in activity levels”. Periodic patterns of space use, as defined here, correspond to the existence of a characteristic, expected time that separates repeated visits to any given area of the home range of the individuals. This is sometimes also called “movement path recursion” [7, 13], albeit the latter phrase does not explicitly convey the notion of a characteristic revisitation time. In contrast, periodicity in activity levels refers to cycles in a derived quantity such as movement speed, turning angle, or in a separately acquired time series such as acceleration, physiological state, or behavioral state. Periodicity in activity levels is expected in most species (rest/activity cycles) and has been the focus of many recent studies [4, 5, 14–19]. Periodic patterns of space use, by contrast, have received little attention so far, possibly due to a lack of an appropriate statistical framework.

In this study, we describe an analytical protocol to uncover periodic patterns of space use in animal tracking data. Animal movement paths (or trajectories, in the vernacular sense of the suite of positions occupied by an individual through continuous time) are the result of multiple co-occurring processes, some stochastic and some deterministic. Periodic patterns in animal tracking data can thereby be obscured by aperiodic noise, i.e., the non-periodic stochastic component of the animal path (not to be confounded with observation noise or telemetry error). The presence of this noise makes it necessary to employ signal processing methods. The proposed protocol in this study is based on periodograms, and in particular the Lomb-Scargle periodogram (LSP) [20]. This periodogram is particularly relevant for movement data because such data often feature missing observations or variation in the duration of sampling intervals (see Methods), a situation in which more standard and widely used periodograms do not apply [20, 21]. First, we introduce and fully describe a new, fast algorithm to estimate the LSP. This new algorithm makes it tractable to analyze long time series (>10^6^ records), for which alternative software would need hours or days of computing time. Second we give a detailed primer about periodicity analysis with periodograms, focusing on the specificities of movement data relative to other uses of periodograms (in astronomy, climate science, physiology, and genetics [22–25]). In particular, we lay out a framework to detect artefactual periodicities created by patterns in the way missing data occur or sampling intervals vary. This issue is commonplace in movement ecology (e.g., if the devices are duty cycled to alternate between fine and coarse sampling rates), but is much less prevalent in other fields and has thus been largely ignored so far. We also emphasize that the background noise of movement data is typically colored, i.e., features temporal autocorrelation in the location, the velocity, or both [26]. This renders existing statistical tests of periodicity (e.g., [27]) inappropriate and requires innovative approaches. Lastly, we highlight the biological insights that can be gained from analyzing periodicity in an animal’s locations instead or in addition to its activity level. We illustrate the approach with three examples from African buffalo (*Syncerus caffer*), waved albatrosses (*Phoebastria irrorrata*), and maned wolves (*Chrysocyon brachyurus*).

## Methods

Periodograms come from the field of signal processing. The approach works by decomposing the signal (here, animal locations) into a sum of sinusoids of fixed frequencies^1^. Periodograms facilitate visual identification of the frequency or frequencies that contribute most to the signal [21], and are the basis for nonparametric statistical tests of periodicity.

### The discrete Fourier transform

The basic issue at hand is to identify periodicity in a signal ***X*** = {***X***_***j***_; *j* = 1, …, *N*} where ***X***_***j***_ is an animal’s recorded location at time *t*_*j*_. In animal movement applications, ***X***_***j***_ is typically two-dimensional (latitude, longitude) but might also have an altitude component. Assuming stationarity, i.e., that the animal does not change its periodic behavior during the course of the study, the discrete Fourier transform of ***X***, denoted ***DFT***{***X***}, yields an estimate of the contribution of any given frequency *f* to the signal [21]:

1 $$ \boldsymbol{D}\boldsymbol{F}\boldsymbol{T}\left\{\boldsymbol{X}\right\}(f)={\displaystyle \sum_{j=1}^N}{\boldsymbol{X}}_j\cdot {e}^{-ijf} $$

with *i* the imaginary unit such that $i=\sqrt{-1}$. Importantly, the DFT requires that the interval Δ*t* between subsequent samples is constant, i.e., *t*_*j*_ = *t*_1_ + (*j* − 1)Δ*t*.

The DFT-periodogram of a multidimensional signal is *P*_*DFT*_(*f*) = 1/*K* ⋅ ∑_*k*_(1/*N* ⋅ |***DFT***{***X***}(*f*)|_***k***_|^2^) where ⋅ |_*k*_ denotes the *k*^th^ dimension (*k* = 1 … *K*). The DFT-periodogram reaches a local maximum if frequency *f* is in phase with the frequency of ***X***. The smallest detectible period in the signal depends on the sampling interval Δ*t*. Frequency *F* = 1/2Δ*t* is known as the Nyquist frequency and is the largest detectible frequency in a signal sampled at interval Δ*t* [21]. There is also a direct relationship between the sample size *N* and the default frequency resolution of the periodogram: Δ*f* = 1/(*N* ⋅ Δ*t*). It is possible to refine the frequency resolution (increase the number of points in the periodogram) up to Δ*f* = *F*/*N*, after which there is no new information in the data to inform new points in the periodogram, leading to autocorrelation in subsequent error terms and an overly smooth periodogram (section A.2 in Additional file 1). The DFT is now a standard tool for signal processing especially since it can be efficiently computed via the fast Fourier transform (FFT) [28], which is available in most statistical software.

### The Lomb-Scargle periodogram: an extension of the DFT important for movement ecology

The condition that the sampling interval is constant, which is necessary to apply the DFT, is rarely met in animal tracking datasets. Tracking datasets are often scheduled to be collected on an even time grid but feature many missing records, or the sampling interval varies in duration due to delays in GPS signal acquisition, or the tracking devices may be purposely “duty cycled” to alternate between coarse and fine sampling rates. This renders the DFT inapplicable without first manipulating the data, which usually involves either thinning them until the sampling intervals are constant, or interpolating missing observations. The Lomb-Scargle periodogram (LSP) [20] extends the DFT to situations with missing data or variable sampling intervals, without ad hoc data manipulations. For this reason, the method has been progressively adopted in many fields where identifying periodicities in imperfect datasets is of interest, including astronomy, climate science, and biology [15–18, 22–24]. This article introduces the LSP as an effective non-parametric method to explore an animal tracking dataset for periodic patterns of space use, something that to our knowledge has not been done previously. Movement ecologists can also use the LSP to look for periodicity in activity level [15–18] but the present study does not emphasize this aspect (see however the section “African buffaloes” below).

### A new algorithm to compute the Lomb-Scargle periodogram

First, we introduce and describe a new algorithm to compute the LSP. The motivation for this new algorithm is to be able to analyze long time series (>10^6^ records like [29]), or large number of moderately long times series (>50 individuals with >10^4^ records per individual like [30]), while maintaining computation times short enough that the protocol can be used at the data exploration stage. Importantly, the choice of an algorithm (our new approach or any of the preexisting ones, see below) does not influence the inference; all LSP algorithms compute the same quantity, but their computation times vary. The full derivation of the new algorithm is described in Additional file 1 and briefly outlined below. First, we define the sampling schedule function *w* on a regular time grid {*t*_*j*_; *j* = 1 … *N*}:

2 $$ w\left({t}_j\right)=\left\{\begin{array}{cc}\hfill 1\hfill & \hfill if\ \boldsymbol{X}\left({t}_j\right)\  is\  recorded\hfill \\ {}\hfill 0\hfill & \hfill if\ \boldsymbol{X}\left({t}_j\right)\  is\  missing\hfill \end{array}\right.\kern2em  with\kern1.5em {t}_{j+1}-{t}_j=\varDelta t\kern0.5em \left(\forall j\right) $$

If the interval between two records was intended to be constant but some records are missing, {*t*_*j*_} simply codes for the intended sampling schedule. In the case of a duty cycle with varying sampling rate, {*t*_*j*_} codes for the finest sampling resolution. If the sampling interval was intended to be constant but eventually varied around that intended value (e.g., due to delays in GPS signal acquisition), our R package ctmm [31, 32] defaults to the median sampling interval for constructing *w* (see further details in section A.7 in Additional file 1). The user can however optionally refine the temporal resolution of {*t*_*j*_} through the res.time option. In this case, we use the Lagrange interpolation of the sinusoids [25] (see more detail below), causing an increase in computing time. We find res.time = 2 to be generally sufficient for most datasets affected by this issue of random variation in sampling interval, while res.time = 4 is almost exact within numerical precision.

Then we exploit the equivalence of the periodogram at frequency *f* to a least-square fit of the data to sinusoids of frequency *f* (Scargle 1982) [20].

3 $$ \boldsymbol{X}\left({t}_j\right)\approx \boldsymbol{A}(f){e}^{2\pi if{t}_j}+\boldsymbol{A}{(f)}^{*}{e}^{-2\pi if{t}_j} $$

where the amplitude ***A***(*f*) is the parameter of interest and the * symbol denotes the complex conjugate. The squared error of this fit is, for each dimension *k*:

4 $$ {L}^{(k)}(f)={\displaystyle \sum_j}w\left({t}_j\right)\cdot {\left|{\left.\boldsymbol{X}\left({t}_j\right)\right|}_{\boldsymbol{k}}-\left({\left.\boldsymbol{A}(f)\right|}_{\boldsymbol{k}}{e}^{2\pi if{t}_j}+{{\left.\boldsymbol{A}(f)\right|}_{\boldsymbol{k}}}^{*}{e}^{-2\pi if{t}_j}\right)\right|}^2 $$

To estimate the amplitude, we minimize the cost function *L*(*f*) = ∑_*k*_*L*^(*k*)^(*f*). It is possible to perform this minimization analytically (Additional file 1; Eqs. A.9–A.11). Following Scargle (1982) [20], the LSP of the *k*^th^ dimension of ***X*** is then expressed as a function of the amplitude:

5 $$ LS{P}^{(k)}(f)=\frac{1}{2}\left({\displaystyle \sum_j}w\left({t}_j\right){\left|{\left.\boldsymbol{X}\left({t}_j\right)\right|}_{\boldsymbol{k}}\right|}^2-\underset{\boldsymbol{A}(f)}{min}\ {L}^{(k)}(f)\right) $$

yielding, after manipulations that are detailed in Additional file 1: Eqs. A13–A15, the following, new formula for the empirical Lomb-Scargle periodogram of a multidimensional time series:

6 $$ \begin{array}{l}L\widehat{S}{P}^{(k)}(f)\Big|=\frac{DFT\left\{w\right\}(0)\cdot \Big|\operatorname{}\boldsymbol{D}\boldsymbol{F}\boldsymbol{T}\left\{w\boldsymbol{X}\right\}(f)\Big|{}_{\boldsymbol{k}}\Big|{}^2-\mathrm{R}\mathrm{e}\left(DFT\left\{w\right\}(2f)\cdot \operatorname{}\boldsymbol{D}\boldsymbol{F}\boldsymbol{T}\left\{w\boldsymbol{X}\right\}(f)\Big|{}_{\boldsymbol{k}}^2\right)}{FT\left\{w\right\}{(0)}^2-\Big|\operatorname{}DFT\left\{w\right\}(2f)\Big|{}_{\boldsymbol{k}}\Big|{}^2}\operatorname{}\kern1em \\ {}\kern1.5em L\widehat{S}P(f)\Big|=\frac{1}{K}{\displaystyle \sum_{k=1}^K}L\widehat{S}{P}^{(k)}(f)\operatorname{}\kern1em \end{array} $$

where Re(⋅) denotes the real part of a complex number and ⋅ |_*k*_ denotes the *k*^th^ dimension of a variable. In Eq. 6, we can see that if there are no missing data, the LSP reduces to the DFT-periodogram of ***X*** because then *DFT*{*w*}(0) = *N* and *DFT*{*w*}(*f*) = 0 (∀ *f* > 0).

**Fig. 1:**
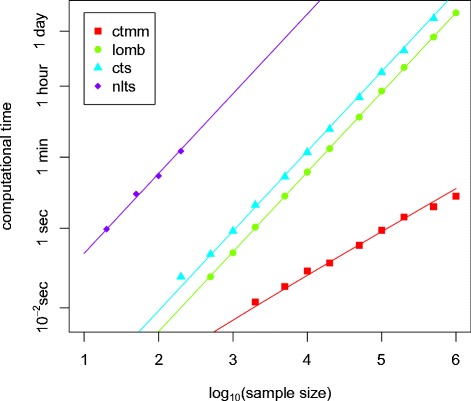
Empirical computing times for the Lomb-Scargle periodogram (LSP) for 2D time series of varying length, using different R-based routines on a 2.5GHZ i5 CPU. All time series featured 50 % randomly selected missing observations, so that the reported sample sizes are half the series’ lengths. “ctmm” corresponds to our new, fast algorithm. “lomb” corresponds to the implementation in the lomb R-package [22], “cts” is from [34], and “nlts” is from [35]. The steeper slope of the “lomb”, “cts”, and “nlts” curves is due to these being based on a *O*(*N* ^2^) algorithm, whereas “ctmm” is based on a *O*(*N* log *N*) algorithm (see main text) and therefore becomes increasingly faster than other R-based alternatives as the sample size increases. The different intercepts for “lomb”, “cts”, and “nlts” indicate a change in per-iteration efficiency across implementations of the same algorithm

What makes this new formula for the LSP fast is that, contrary to the original implementation by Scargle (1982) [20], it is entirely based on the DFT, and can therefore be computed using the fast Fourier transform (FFT) [28]^2^. Exploiting the proven speed of a known algorithm like the FFT is a standard method for developing more efficient computing techniques (e.g., [33]). For the case that interests us here, we assessed the gain in computing time using both algorithmic complexity considerations, and by directly measuring computing time. The computational complexity of an algorithm is conveyed with notation *O,* meaning “proportional to”. The complexity of the FFT algorithm is known to be *O*(*N* log *N*) [28], that is, the computing time of the FFT is proportional to *N* log *N*, where *N* is the number of records in the time series. Being entirely based on the FFT, our new algorithm for the LSP is therefore also *O*(*N* log *N*). By contrast, Scargle’s original computation of the LSP requires running twice through the time series, yielding a *O*(*N*^2^) complexity, that is a computing speed proportional to *N*^2^. The difference between *O*(*N* log *N*) and *O*(*N*^2^) can be enormous in practice: our algorithm with *O*(*N* log *N*) complexity runs within seconds in situations where algorithms with *O*(*N*^2^) complexity need more than a day (Fig. 1). Previously, only Press & Rybicki managed to derive an *O*(*N* log *N*) routine for the LSP, also by using the FFT [25]. However, the Press & Rybicki algorithm is much more complex than ours; as a consequence, all R packages and freeware for biologists that have a LSP functionality, and of which we are aware [22, 34–37], are based on Scargle’s original algorithm, not on Press & Rybicki’s fast algorithm. Furthermore, although the Press & Rybicki algorithm is based on the FFT like our new algorithm, it requires additional calculations including finer gridding, Lagrange interpolation, and *O*(*N*) square-root and trigonometric function evaluations [25], that our approach does not require as long as the time grid {*t*_*j*_} is even or can be approximated as such (see beginning of section for definition of {*t*_*j*_}). These additional steps also render the Press & Rybicki algorithm only an approximation to the LSP, whereas our approach is exact as long as the time grid {*t*_*j*_} is even. When the interval durations are variable, i.e., {*t*_*j*_} is not even, both methods are approximate. In the ctmm implementation of our fast algorithm, users can further control the amount of error through the frequency resolution option (res.freq).

In summary, because it is based on the FFT and is therefore of *O*(*N* log *N*) complexity, our new algorithm substantially increases the speed of the computation of the LSP compared to other R implementations, which are based on Scargle’s original *O*(*N*^2^) algorithm (Fig. 1). This makes LSP computation tractable even for long time series (like [29]), or for numerous time series (like [30]). Importantly, whatever the algorithm used (ours, Press & Rybicki’s, or Scargle’s), the biological inference and statistical power are not affected: the same quantity is estimated.

Lastly, our implementation in the R package ctmm [31, 32] also accommodates two important features that are commonplace in movement data but infrequent in other fields. The first of these features is multi-dimensionality; all other LSP implementations in R are 1-dimensional and therefore require post-hoc manipulations to combine results from latitude and longitude time series. In the ctmm implementation, periodicity in the latitude, longitude, and altitude can be investigated jointly or separately. The second feature is multiple individuals. Movement ecology datasets often comprise multiple individuals which are expected to behave in similar ways, i.e., exhibit the same periodic patterns. In such a situation, ctmm allows fitting multi-individual periodograms, in order to augment sample size and better separate the periodic patterns from the background noise. The principle is described in section A.3 of Additional file 1 and an example presented in the “Maned wolves” section below.

### Interpreting periodograms

**Fig. 2:**
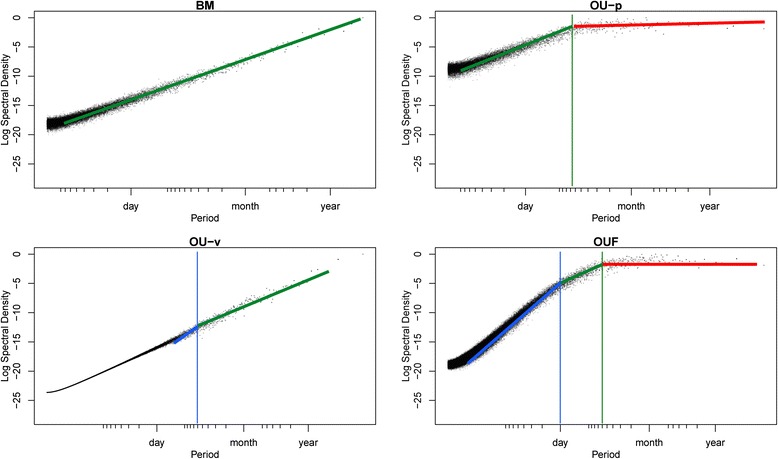
Empirical Lomb-Scargle periodogram (LSP) of four standard, aperiodic continuous-time stochastic movement models, showing the expected increase with period, and the rough aspect due to the stochastic nature of the process. “BM” is Brownian motion, “OU-p” is Ornstein-Uhlenbeck position process, “OU-v” is Ornstein-Uhlenbeck velocity process (a.k.a. Integrated Ornstein-Uhlenbeck process), “OUF” is Ornstein-Uhlenbeck position process with foraging, i.e., a combination of autocorrelation in both the velocity and the position of the animal. These four models are described in full detail in [26]. Theoretical periodogram values are plotted with solid lines. Green indicates a slope of 2, blue a slope of 4, and red a slope of 0. The vertical lines represent expected characteristic time scales separating two regimes of the periodogram. These time scales are equal to 2*πτ* where *τ* is the characteristic autocorrelation time of the underlying models used to generate the data. The flattening of the curves near the Nyquist frequency (small period values) is an unwanted characteristic of all periodograms, that makes periodicity inference unreliable near the Nyquist frequency

First, the overall shape of the periodogram of an animal’s track record is influenced by the temporal autocorrelation structure of the aperiodic background noise. This issue was previously largely ignored because in astronomy, which is where the LSP was developed and where most extensions of the LSP have been published, the background noise can be assumed to be independently and identically distributed (“white”) [27]. However, in movement ecology, the stochastic aperiodic component of the animals’ paths is expected to exhibit temporal autocorrelation in the location process, the velocity process, or both (“colored”) [26]. As a consequence, previously developed statistical tests of periodicity [22, 27] cannot be applied to movement data. Instead, the peaks in the periodograms must be compared to the autocorrelated background noise (see the section entitled “Artefactual periodicities and a new statistical test for periodic patterns of space use” below). A graphic representation of the LSP of the four most commonly used aperiodic continuous-time stochastic movement models is provided in Fig. 2. We do not recommend using the LSP as a diagnostic tool to distinguish different aperiodic stochastic models (see instead [26, 32, 38]). However, knowing what the periodogram of an aperiodic animal path should look like can be very useful to interpret empirical periodograms, especially given the rough aspect of most empirical periodograms.

**Fig. 3:**
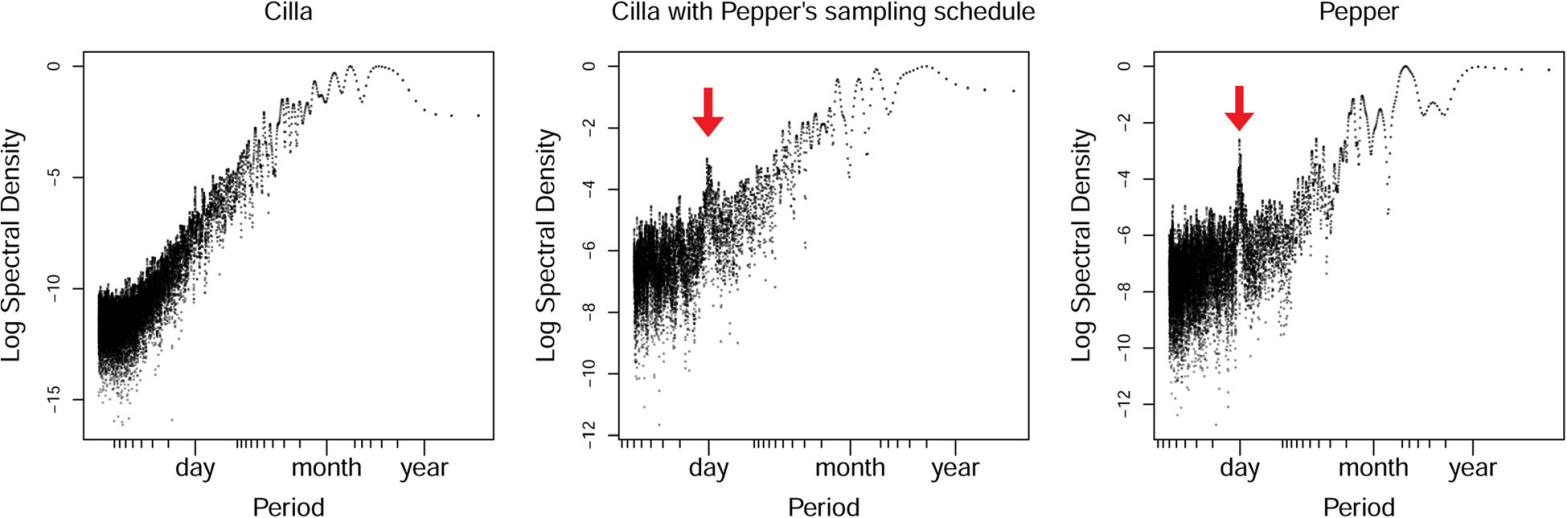
*Left*: Periodogram of buffalo Cilla’s tracking data under the original sampling schedule. *Center*: Periodogram of Cilla’s data when resampled to mimic Pepper’s sampling schedule. *Right*: Periodogram of Pepper’s tracking data. The arrows point at the peaks corresponding to the one-day period

**Fig. 4:**
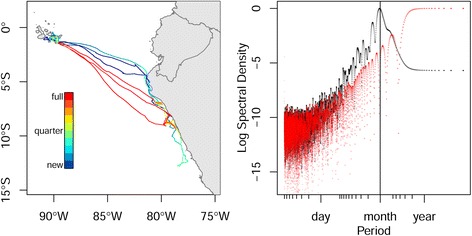
Tracking data from one waved albatross. *Left*: plotted with color scale indicating moon phase. Blue colors are close to the new moon and red colors to the full moon. *Right*: periodograms. The black periodogram is from the tracking data and the red periodogram is from the sampling schedule. The presence of a peak in the black periodogram for a period of one lunar cycle (vertical line), and its absence in the red periodogram, indicate that the periodic pattern is not caused by the sampling schedule

**Fig. 5:**
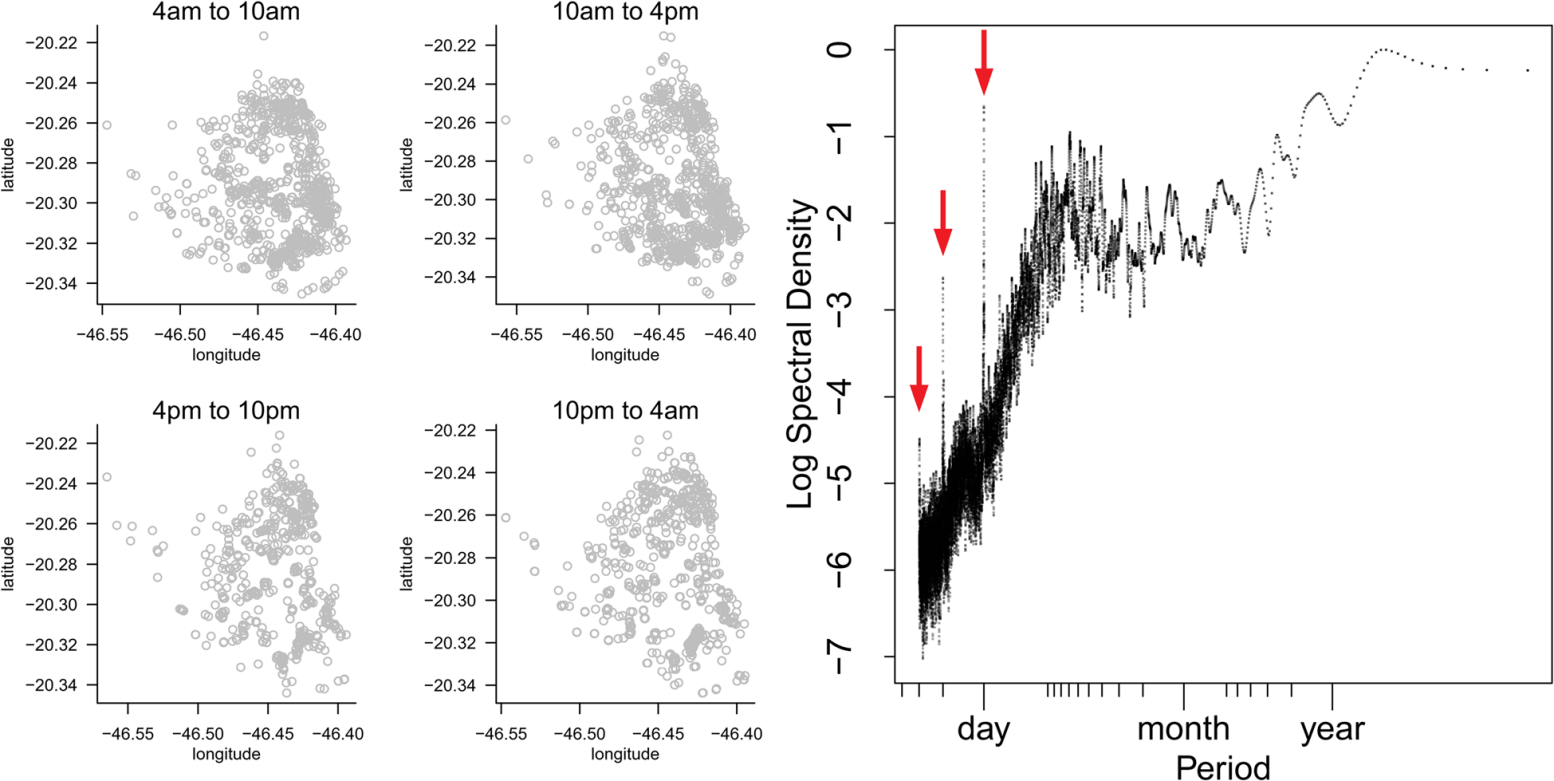
Tracking data from maned wolves. *Left*: plotted for one individual (Amadeo) according to time of the day, to show the difficulty to visually detect the periodic pattern because of the important stochastic component in the movement process. *Right*: multi-individual periodogram from Amadeo and 7 other wolves, showing the one-day periodicity and the associated harmonics series (red arrows)

The important features to look for in periodograms are peaks (Additional file 2, section C1). Peaks correspond to frequencies that resonate with the signal. These peaks vary in width and height (Figs. 3, 4 and 5; Additional file 2). Regarding the width of the peaks, as mentioned earlier, the default resolution of the periodogram is, in the frequency domain, *Δf* = 1/(*N* ⋅ *Δt*) where *N* is the number of recorded locations and *Δt* is the (median) sampling interval between two records. In the time domain, this yields a resolution that increases with the period: *ΔT* = *T*^2^/(*N* ⋅ *Δt*) ≈ *T*, meaning that, if the sampling schedule stays the same, the longer the period, the wider the peak. Peak width, therefore, is typically an artifact of the periodogram’s natural resolution, so that longer periods will often have wider peaks (Additional file 2, section C.4). Peak width thereby typically carries little biological information about periodicity in the mean of the movement process. Some of this artefactual variation in peak width can be removed using the max = TRUE option of the plot method for periodogram objects in ctmm, and by increasing the frequency resolution (the number of points) with the res.freq option when computing the periodogram. With the max=TRUE option, only local maxima of the periodogram are plotted. This post-hoc coarsening of the frequency resolution removes in particular the artefactual oscillations caused by the autocorrelation in the error term of the periodogram with a period of 1/*D* on the frequency scale (∼ *T*^2^/*D* on the period scale), where *D* is the overall study duration. These oscillations, when not discarded using the max option, are typically visible for large periods (Figs. 3, 4 and 5).

By contrast, the height of the peaks, i.e., the difference between the periodogram value at the peak and around the peak, conveys information about the signal-to-noise ratio [22, 27]. The statistical significance of the periodic pattern can be inferred by comparing the peak height to the baseline periodogram of the background noise [27] (see more details under “Artefactual periodicities and a new statistical test for periodic patterns of space use” below).

Third, periodogram interpretation needs to take harmonic series into account (section C.2 in Additional file 2). A harmonic is defined as a component frequency of the signal that is an integer multiple of the fundamental frequency (the frequency with the largest signal-to-noise ratio). Harmonic series occur if the periodic signal is not perfectly sinusoidal, i.e., if the waveform is different from a sinusoid, or if the repeated pattern is different from an ellipse. In movement ecology, periodic patterns of space use are therefore expected to always generate a series of harmonics. We performed a simulation study to illustrate the occurrence of harmonics in a few simplified situations (section C.2 in Additional file 2). These simulations illustrate that users should not over-interpret peaks in the periodogram that occur for periods that are a fraction of the fundamental period (the highest peak in the periodogram). Even in very simplified cases, predicting which harmonics would be activated was far from straightforward, and depended on the sampling schedule, the velocity, and the shape of the repeated pattern. In real-life applications, harmonic series are unlikely to bring interpretable information about the shape of the repeated pattern. The difficulty further lies in identifying whether the signal is multi-periodic (e.g., both daily and hourly periods) or mono-periodic with harmonics. If peaks occur for periods that are not integer multiples of each other, the signal is multi-periodic. If the peak for a short period (e.g., one hour) is higher than the peak for a long period (e.g., one day), the signal is also multi-periodic. In most other situations, the signal is likely monoperiodic with harmonics.

### Artefactual periodicities and a new statistical test for periodic patterns of space use

The issue at hand in this section is the identification of peaks in the periodogram that are not related to an underlying periodic behavior, but are instead caused by the sampling schedule. This can for example be the case if the tracking device is duty cycled to alternate between fine and coarse sampling rates, in effect creating a periodic sampling schedule, or if there is autocorrelation in the way missing data occur. In such situations, periodicity in the sampling schedule can be transferred into periodicity in the animal tracking record, even if the true, underlying path is not periodic (section C.3 in Additional file 2). In most previous applications of the LSP, this issue was largely ignored: researchers assumed missing observations or sampling interval variations occurred without autocorrelation [22]. In movement ecology, the issue cannot be ignored anymore (e.g., [26]), requiring innovative treatments.

In order to jointly deal with the issues of 1) temporally autocorrelated background noise; 2) possible confusion of random peaks with periodicity-induced peaks; and 3) artefactual periodicities created by the sampling schedule, we devised the following two-pronged approached based on a null model simulation routine and a visual diagnostic.

#### Visual diagnostic

The objective here is to visually compare the periodogram of the location time series, ***X***(*t*), and the periodogram of the sampling schedule, *w*(*t*). If the periodicity observed in the data is artefactual, then the same peak should occur in the periodogram of the sampling schedule and in the periodogram of the locations. After rescaling the two periodograms so that they both have a maximum value of zero (automatically performed by ctmm), the periodogram of the recorded locations is plotted alongside the periodogram of *w*. If a peak occurs in both periodograms for the same period, then it is likely that the detected periodicity is artefactual. However, a true periodicity could plausibly be superimposed on an artefactual one, so that the occurrence of a peak of same periodicity in the two periodograms does not prove the absence of a periodic pattern, but only indicates the risk of false positive due to issues in the sampling schedule. This visual diagnostic approach has the advantage of being fully non-parametric. It can thus be employed on any time series irrespective of which type of underlying movement process generated the data or how irregular the sampling schedule is. It can therefore be used without restriction. The visual diagnostic approach is available in package ctmm through the diagnostic = TRUE option of the plot method for periodogram objects.

#### Null model approach

Here, we first use an information-theoretic approach to select a preferred aperiodic continuous-time stochastic model and fit it to the data. The theory for these models is developed in e.g., [38] and recommendations for model fitting and model selection with ctmm are detailed in [32]. The preferred aperiodic continuous-time stochastic model acts as the null model, representing the hypothesis “there are no periodic patterns of space use in the data”. It is the reference frame against which peaks in the periodogram are going to be compared. We use this model to generate simulated datasets with the same sampling schedule as the real data. Any irregularity in the sampling schedule or pattern in the way missing data occur is thus carried over in the simulations. Finally, we compute the proportion of the simulated periodograms in which the value at the period of interest is larger than the value from the real data. This proportion is the *P*-value of the periodicity test (the sensitivity of the test depends on the number of simulations). An analogous approach was proposed for the case where the background noise is independently and identically distributed (“white”) by [27] and is implemented by default in the lomb package [22]. Here, we thus generalize this original statistical test of periodicity to the case where the background noise is temporally autocorrelated (“colored”), which will typically be the case with movement data. An R script implementing this approach (assuming a preferred null model has already been selected) is provided in Additional file 3.

### African buffaloes

This case study is aimed at 1) illustrating the issue of artefactual periodicities created by the sampling schedule (see also section C.3 in Additional file 2 for a simulated example), and 2) highlighting that periodicities in activity levels need not be translated into periodic patterns of space use. Data came from two African buffalo cows named Cilla and Pepper in the Kruger National Park of South Africa, with hourly GPS position records [39]. Cilla had no missing observations and displayed aperiodic space use, whereas Pepper had many missing observations and a periodogram that exhibited a clear daily periodicity. Based on the visual diagnostic, Calabrese et al. [32] suspected that the periodicity in Pepper’s periodogram was artefactual and caused by a collar malfunction. To prove that point, we discarded data points from Cilla in order to mimic Pepper’s sampling schedule. To do so, we first shifted the sampling schedules so that the time series for the two individuals started at the same time, and then discarded all fixes from Cilla’s tracking record that were collected more than 10 min from a fix from Pepper. This yielded a subsample of Cilla’s locations affected by the same missing data problem as Pepper. We predicted that the periodogram from Cilla’s resampled tracking record would exhibit the same (artefactual) peak as Pepper’s. Sample sizes were 3528 fixes for Cilla, of which 2153 were discarded in the manipulation, and 1726 fixes for Pepper. Lastly, we tested Pepper’s periodicity using the above-described null-model approach. If artefactual, the periodicity should not be significant in this test (*P*-value >0.05).

We also used Cilla’s (complete) data to illustrate the difference between periodic patterns of space use and periodicity in activity level. The former corresponds to the existence of a characteristic revisitation time between two passages of an animal in a given place, and is our focus in this paper. The latter corresponds to rest/activity cycles, and has been the focus of most other studies applying periodograms to movement data [4, 14–18]. In this case, African buffaloes characteristically show reduced activity during the mid-day heat, so we predicted a daily periodicity in Cilla’s activity level whether or not her space use was periodic. Activity was measured by movement speed, which we quantified as the length of each recorded step divided by the duration of the corresponding time interval.

### Waved albatrosses

We analyzed 90-min resolution GPS tracking data from waved albatrosses during their 2008 breeding season [40, 41]. We selected data from four adult birds breeding on Española island in the Galapagos archipelago, that were monitored for more than 45 days (range 47–97 days) and undertook more than one fishing trip during that time. During incubation, the male and female of a pair alternate nest duties and fishing trips, so that the nest is always attended [40]. Most fishing trips are towards nutrient-rich up-welling zones off the coast of Peru about 1200 km from the breeding island. Waved albatross’ diet include a large proportion of squids [42] which are mainly available to them at night [6]. Although waved albatrosses exhibit some morphological adaptations such as larger eyes and larger distance between them than related species, we suppose that they still have poor night vision like most other albatrosses [43, 44]. Hence, we formulate the hypothesis that foraging behavior should be constrained by moon light, and therefore exhibit a periodicity of c. 29.5 days.

### Maned wolves

Maned wolf, the largest South American canid, is exceptional among similarly-sized canids for its solitary foraging behavior and omnivorous diet, which raises questions regarding its daily energy budget [45] and social system [46]. The occurrence of a periodic pattern of space use corresponding to “routine behaviors” is potentially of foremost importance for energy budget and sociality, but remains poorly understood. While there is anecdotal evidence that maned wolves repeatedly use the same paths when patrolling their home range in search of food in Brazil [47], routes are diversified in Bolivia [48]. Interestingly, the behavior is documented in other, more intensively-studied canid species, but even in these species the extent to which it is expressed is variable and poorly known [49, 50]. We used tracking data from eight wolves collected in or near the Serra da Canastra National Park, Brazil, a grassland ecosystem, and used the LSP methodology to test for a daily periodicity in those eight individuals. Maned wolves were captured using live-traps baited with cooked chicken and sardines, sedated (with direct injection of tiletamine-zolazepan), and equipped with VHF/GPS-Collars (Lotek Wireless Inc. GPS 3300S and Iridium Track 1D, and Sirtrack Limited Pinnacle Lite G5C 275D). The devices were set to record one location every 1 to 4 h (depending on the animal).

## Results

### African buffaloes

The LSP of Cilla’s locations was characteristic of an aperiodic random walk (no peak), whereas the LSP of Pepper’s locations suggested a daily cycle (Fig. 3). When applying Pepper’s pattern of missing observations to Cilla’s location time series, we retrieved, as predicted, a signal of daily periodicity (Fig. 3). This illustrated that the periodic pattern was created by autocorrelation in the way observations were missing, not the animals’ behavior. The *P*-value of 0.29 from 150 simulations in the null model test applied to Pepper’s locations confirmed that the observed periodic pattern in Pepper’s tracking record was artefactual.

When applying the LSP to Cilla’s speed record (vs. location record previously), we found, as predicted, a significant daily periodicity (*P*-value <0.01 from 150 simulations; Additional file 2: Figure C5). Cilla’s *activity* was thus periodic even though her *space use* was not (Fig. 3). This illustrates how periodic patterns of space use are decoupled from periodicity in activity levels in this species.

### Waved albatrosses

All four albatross individuals exhibited a significant periodicity (all *P*-values <0.005 from 1000 simulations each) with estimated period ranging from 22 to 35 days. In the individual that undertook the longest trips, reaching waters 1800 km from its breeding colony with an estimated periodicity of 29 days, the association with moon phase was very pronounced (Fig. 4). During three monitored cycles, the bird left the colony shortly before the new moon and started its return journey shortly after the full moon, presumably so that both it and its mate could forage during periods with some moon light.

### Maned wolves

The one-day periodicity was statistically significant for all eight individuals (all *P*-values <0.05 from 1000 simulations each; Fig. 5, right panel), even if the periodic pattern of space use was not directly evident in the raw tracking data (Fig. 5, left panel). In other words, those eight wolves showed a significant tendency to patrol their home range along daily-repeated routes, but this pattern was obscured by the important background noise (the aperiodic stochastic components of the paths).

## Discussion

In this study, we introduced an analytical protocol based on the Lomb-Scargle periodogram (LSP) to facilitate exploration of animal tracking datasets for periodic patterns, even if those datasets feature missing observations or variation in the duration of sampling intervals. We described a new, fast algorithm to compute the LSP. The gain in speed makes it tractable to quickly analyze large datasets (>10^6^ records) that would otherwise require days of computing time with other R packages. We also gave a detailed primer about periodicity analysis, focusing on the specificities of movement data. In particular, we described a methodology to identify artefactual periodicities created by the sampling schedule, an issue that is commonplace in animal tracking data but much rarer in other uses of the LSP. We developed a statistical test of periodicity that applies when the background noise is temporally autocorrelated, which is typically the case in movement data, and that is robust to irregularities in the sampling schedule. We also accommodated multidimensionality (2D, 3D) and the possibility that several animals exhibit the same type of periodic patterns, both features being commonplace in movement datasets. We highlighted that novel biological insights can be gained from analyzing periodicity in the *locations* of the animals, not (only) in their *activity* levels, as is more commonly done. Applying our LSP-based analytical protocol to location data from three species revealed that an ungulate exhibited periodicity in its movement speed but not in its locations, that a central place forager tracked moon phase, and that the paths of a range-resident canid included a daily patrolling component that was initially masked by the stochasticity of the paths.

### On the use of periodograms in movement ecology

As outlined above, the study of periodic behaviors can be based on the animals’ locations or on their activity levels, or both. In all cases, the LSP represents the best available non-parametric tool to detect periodic patterns in imperfect data [22, 24]. Periodicity in activity level and periodic patterns of space use represent two distinct phenomena, as the case of African buffalo Cilla illustrates. Cilla showed no sign of periodicity in her locations, i.e., no periodic pattern of space use (Fig. 3), but a highly significant one-day periodicity in her movement speed, i.e., statistically significant rest/activity cycles (Additional file 2: Figure C5). Rest/activity cycles are expected to occur in almost all species [4, 14–19], but see [5]. By contrast, periodic patterns of space use are expected to be much less widespread, but likely to be of fundamental biological interest when and where they do occur. Periodic patterns of space use are indeed only expected under specific conditions, such as when breeding and foraging habitats are distinct [51], or when forage quality and predation risk are positively correlated [13], or when being predictable allows avoiding direct contacts with neighbors and therefore reduces the potential for territorial conflicts [10]. The few previous studies that focused on periodicity in animals’ locations first defined an area of interest and then transformed the tracking record into a series of Booleans representing presence, entry, or exit from that area of interest [13, 52]. This procedure restricts the inference to a specific area of interest that the researcher a priori designates as likely to be revisited periodically. The LSP allows researchers to avoid making such a priori decisions, and is thus a more broadly applicable data exploration tool.

### Semivariogram or periodogram?

Temporal autocorrelation, of which periodic patterns are a particular form, is typically investigated using semivariograms [26, 53]. Semivariograms could therefore theoretically be used for inference about periodicity (Additional file 2, section C.4). However, the empirical semivariogram often suffers strong autocorrelation in its error term, and is not by construct negative definite [53], which can lead to flawed inference about periodicity when the data are noisy and finely sampled. By contrast, as long as the minimum frequency resolution *F*/*N* is not exceeded, the empirical periodogram is largely without autocorrelation in its error term, and is by construct always positive [21]. As a side note, the absence of autocorrelation in the error term is also the cause for the rough appearance of most empirical periodograms. In addition, the periodogram is constructed in the frequency domain, which makes visual diagnosis much more straightforward, especially in the presence of multiple periodicities in the data. When the data are relatively coarsely sampled relative to the period of the periodic pattern of space use, the semivariogram can nevertheless yield a useful confirmation of a pattern that might be hard to detect in the periodogram because of the width of the peak (Additional file 2, section C.4). Lastly, neither periodograms nor semivariograms accommodate regime shifts or non-stationary dynamics, which have been a topic of interest recently [4, 14, 19]. In other words, current versions of empirical periodograms and semivariograms average over non-stationarity and allow users to visualize the time-averaged processes.

## Conclusions

In conclusion, we recommend the Lomb-Scargle periodogram (a fast implementation of which is available in the R-package ctmm) as a non-parametric, exploratory tool to explore animal tracking datasets for periodic patterns of space use and other periodic patterns such as periodicity in activity level. The often used Discrete Fourier Transform (DFT) requires constant time intervals between subsequent locations records, a condition that is rarely met in animal tracking datasets and therefore forces users to perform ad hoc data manipulations before analysis. The LSP is strictly equivalent to the DFT-periodogram if the time intervals are constant, and does not require ad hoc data manipulation if the time intervals are not constant. We confirm however that both approaches are challenged when sampling intervals vary in a strongly temporally autocorrelated way. Using any periodogram blindly in this case can lead to the detection of artefactual periodicities. Diagnostic tools presented in this study can be used to identify artefactual periodicities created by the sampling schedule, so that further analyses can be adapted accordingly. As animal tracking datasets become longer and finer-resolution, and more individuals can be tracked for the same budget, we envision increased interest in exploratory non-parametric methods like the periodogram, which pave the way for case-tailored parametric tests and movement models.

## Abbreviations

DFT, discrete Fourier transform; FFT, fast Fourier transform; GPS, global positioning system; LSP, Lomb-Scargle periodogram; OU, Ornstein-Uhlenbeck process

## Additional files

Additional file 1: The Lomb-Scargle periodogram in movement ecology. (PDF 229 kb) Additional file 2: Simulation study. (PDF 581 kb) Additional file 3: R code for a statistical test of periodicity in animal tracking data. (R 1 kb)

## Footnotes

1 As a reminder, the frequency of a signal is the inverse of its period.

2 In the ctmm implementation, we use the fftw R package, which is a wrapper around the FFTW C-library [54], and is faster and more robust than R's built in FFT routines. By default, we inflate the frequency resolution until the grid size is a power of two, to avoid slowing down the FFT. This excess frequency resolution can be removed with the max = TRUE option of our plot method for periodogram objects.
