# Supplementary material for: Uncovering periodic patterns of space use in animal tracking data with periodograms, including a new algorithm for the Lomb-Scargle periodogram and improved randomization tests
Source: Mov Ecol. 2016 Aug 1;4:19. doi: 10.1186/s40462-016-0084-7 (PMC4968009; doi:10.1186/s40462-016-0084-7)
Supplement: Additional file 1: — The Lomb-Scargle periodogram in movement ecology. (PDF 229 kb) [file 40462_2016_84_MOESM1_ESM.pdf]

Appendix to "Periodograms for movement ecologists: a non-parametric method to uncover periodic patterns of space use in animal tracking data"

Guillaume Péron[1], Chris H. Fleming[1,2], Rogerio C. de Paula[1], and Justin M. Calabrese[1,2]

[1]Conservation Ecology Center, Smithsonian Conservation Biology Institute, National Zoological Park, 1500 Remount Rd., Front Royal, VA 22630
[2]Department of Biology, University of Maryland College Park, College Park, Maryland 20742
[3]National Research Center for Carnivore Conservation (CENAP/ICMBio), Atibaia, Sao Paulo, Brazil

April 29, 2016

Appendix A: FFT Lomb-Scargle periodogram

A periodogram estimates the spectral-density function $\tilde{\sigma}(f)$ at frequency f, which is itself the Fourier transform of the stationary (or time-averaged) autocorrelation function $\sigma(\tau)$.

$$\tilde{\sigma}(f) \equiv \int_{-\infty}^{+\infty} dt\, e^{-2\pi\imath f\tau}\, \sigma(\tau) \propto \mathrm{VAR}\left[\int_{-\infty}^{+\infty} dt\, e^{-2\pi\imath f t}\, x(t)\right] = \mathrm{VAR}[\tilde{x}(f)]\ , \tag{A.1}$$

$$\sigma(\tau) = \underbrace{\lim_{T\to\infty} \frac{1}{2T} \int_{-T}^{+T} dt}_{\text{time average}}\, \mathrm{COV}[x(t+\tau), x(t)]\,, \tag{A.2}$$

where the noted time average is unnecessary for process with stationary autocorrelation functions that have no dependence upon absolute time t, and where we define the variance of any complex-valued process $\mathrm{VAR}[z] \equiv \langle z\, z^* \rangle$, where z^* denotes the complex conjugate of z. The DFT

periodogram is a straightforward approximation to the variance of the (continuous) Fourier transform of $x(t)$ via the discrete Fourier transform (DFT). Another way of motivating the periodogram, which more readily generalizes in the case of missing data, is that the periodogram at frequency f is equivalent to the the 'power' derived from a least-squares fit of all n data points to a sinusoid of frequency f. "Power" in this context is bit of a mathematical abstraction that we will later define, but note that if the signal $x(t)$ represents oscillator position, velocity, electrical current, or electrical voltage, then the signal's variance $\sigma(0)$ is proportional to its average oscillator potential, kinetic, inductor, or resistor energy, respectively. Further note that with Parseval's theorem we can distribute this "energy" among times or frequencies

$$\sigma(0) \propto \int_{-\infty}^{+\infty} dt \, \mathrm{VAR}[x(t)] = \int_{-\infty}^{+\infty} df \, \mathrm{VAR}[\tilde{x}(f)] \, . \tag{A.3}$$

Therefore the periodogram estimates autocorrelation structure via the spectral-density function and distributes the signal's variance among frequencies in a way that is conjugate to how the variance is distributed among times.

The naive implementation of the DFT or LS periodogram has a computational cost of $\mathcal{O}(n^2)$ to estimate the n most relevant frequencies within the range $df \leq f \leq F$ (Table A.1), where df is the natural frequency resolution of the data and F is the Nyquist frequency or natural frequency cutoff of the data. The fast Fourier transform (FFT) reduces this computational cost to $\mathcal{O}(n \log n)$ for the DFT periodogram. Furthermore, Press and Rybicki (1989) discovered that, after expanding the sinusoids in an evenly spaced grid via Lagrange interpolating polynomials, the LS periodogram can also be calculated using FFT techniques. As the Lagrange interpolants are approximations of the true functions, the evenly-spaced grid needs to be fine enough to accurately capture the high-frequency behavior. Our implementation of the LS periodogram differs in that it is given by a simple expression without the necessity of Lagrange interpolation. In the case of evenly scheduled data, where our implementation is exact, the cost savings of avoiding Lagrange interpolation are

typically on the order of a factor of 10-40 (Press and Rybicki, 1989).

Time domain		Frequency domain
temporal resolution<br>dt	$\Leftrightarrow$	frequency range<br>$F = 1/(2dt)$
temporal range<br>T	$\Leftrightarrow$	frequency resolution<br>$df = 1/T$

Table A.1: Conjugate relationship between the temporal quality of data and the frequential quality of data under the DFT. These relations are still approximately true for the Lomb–Scargle periodogram (LSP).

For our derivation, we exploit the fact that for the realization of the process of interest $x(t)$, we know the indicator function

$$w(t) \equiv \begin{cases} 1\,, & x(t) \text{ observed} \\ 0\,, & x(t) \text{ missed} \end{cases}\,, \tag{A.4}$$

and, though we have not always measured $x(t)$ on a uniform time grid, we have measured $w(t)$ and (effectively) $w(t)\,x(t)$ on a uniform time grid. This scheme is natural for data that are evenly scheduled but feature missing values, as the observed times neatly reside in a uniformly spaced grid of scheduled observation times that is only slightly larger than the number of observations.

Our fast implementation of the LS periodogram then follows the derivation of Scargle (1982). We exploit the equivalence of the LS periodogram to a least-squares fit of the data to independent sinusoids

$$x(t) \approx x_f(t) = A(f)\, e^{+2\pi\imath f t} + A(f)^*\, e^{-2\pi\imath f t}\,, \tag{A.5}$$

to obtain the amplitudes $A(f)$. This fitting has the associated least-squares cost function

$$L(f) = \sum_t w(t)\,|x(t) - x_f(t)|^2\,, \tag{A.6}$$

given weights $w(t)$. Solutions for the amplitudes must therefore satisfy the system of equations

$$\begin{bmatrix} \sum_t w(t) & \sum_t e^{-4\pi\imath ft}\, w(t) \\ \sum_t e^{+4\pi\imath ft}\, w(t) & \sum_t w(t) \end{bmatrix} \begin{bmatrix} \hat{A}(f) \\ \hat{A}(f)^* \end{bmatrix} = \begin{bmatrix} \sum_t e^{-2\pi\imath ft}\, w(t)\, x(t) \\ \sum_t e^{+2\pi\imath ft}\, w(t)\, x(t) \end{bmatrix}. \tag{A.7}$$

If we represent the data $x(t)$ and weights $w(t)$ on a uniform time grid, with missing data naturally weighted by zero, then all of these sums can be calculated with the FFT implementation of the DFT of the weights $w(t)$

$$\mathrm{DFT}\{w\}(f) = W(f) = \sum_t e^{-2\pi\imath ft}\, w(t)\,, \tag{A.8}$$

so that we have

$$\begin{bmatrix} W(0) & W(2f) \\ W(2f)^* & W(0) \end{bmatrix} \begin{bmatrix} \hat{A}(f) \\ \hat{A}(f)^* \end{bmatrix} = \begin{bmatrix} \mathrm{DFT}\{wx\}(f) \\ \mathrm{DFT}\{wx\}(f)^* \end{bmatrix}. \tag{A.9}$$

For frequencies f larger than half the Nyquist frequency F, it is convenient to exploit the periodicity of the transform

$$W(2f) = W(2f - 2F)\,. \tag{A.10}$$

Solving Eq. (A.9) for the amplitudes, we then have

$$\hat{A}(f) = \frac{W(0)\,\mathrm{DFT}\{wx\}(f) - W(2f)\,\mathrm{DFT}\{wx\}(f)^*}{W(0)^2 - |W(2f)|^2}\,, \tag{A.11}$$

while the power estimate in Scargle (1982) Eq. (C3) equates to

$$\hat{P}(f) = \frac{1}{2}\left(\sum_t w(t)\, |x(t)|^2 - \min_{A(f)} L(f) \right)\,, \tag{A.12}$$

by expressing his power estimate in terms of both $x(t)$ and $w(t)$. Refer to Scargle (1982) for more details on the "power", but note simply that it measures the amount of variability in the data attributable to frequency f. Combining equations (A.12), (A.6), and (A.5), we now have

$$\begin{aligned}
\hat{P}_{\mathrm{LSP}}(f) &= -\sum_t w(t)\, x(t)\, \hat{x}_f(t) + \frac{1}{2}\sum_t w(t)\, \hat{x}_f(t)^2\,, && \text{(A.13)}\\
&= -\,\hat{A}(f)\sum_t e^{+2\pi\imath f t}\, w(t)\, x(t) - \hat{A}(f)^*\sum_t e^{-2\pi\imath f t}\, w(t)\, x(t) && \text{(A.14)}\\
&\quad + \frac{1}{2}\hat{A}(f)^2\sum_t e^{+4\pi\imath f t} w(t) + \left|\hat{A}(f)\right|^2\sum_t w(t) + \frac{1}{2}\hat{A}(f)^{2*}\sum_t e^{-4\pi\imath f t} w(t)\,,\\
&= -\hat{A}(f)\,\mathrm{DFT}\{wx\}(f)^* - \hat{A}(f)^*\mathrm{DFT}\{wx\}(f) && \text{(A.15)}\\
&\quad + \frac{1}{2}\hat{A}(f)^2\, W(2f)^* + \left|\hat{A}(f)\right|^2 W(0) + \frac{1}{2}\hat{A}(f)^{2*}\, W(2f)\,,
\end{aligned}$$

and then combining this expression with Eq. (A.11) we have after some simplification

$$\hat{P}_{\mathrm{LSP}}(f) = \frac{W(0)\,|\mathrm{DFT}\{wx\}(f)|^2 - \mathrm{Re}\big[W(2f)^*\,\mathrm{DFT}\{wx\}(f)^2\big]}{W(0)^2 - |W(2f)|^2}\,, \tag{A.16}$$

which can be constructed entirely from the FFT of $w(t)$ and $w(t)\,x(t)$.

A.1 Comparison to the DFT periodogram

Strictly speaking the DFT periodogram is not defined for missing data, but researchers not familiar with the Lomb-Scargle periodogram will typically replace a few missing values of the data with the mean or an interpolated value. In the case of interpolating the missing value, the effect is to bias the autocorrelation estimate with the properties of the interpolating function at the scale of the gaps. I.e., straight-line interpolation over gaps of width Δt gives the appearance of a more ballistic process over timescales $\lesssim \Delta t$ and frequency scales of $\gtrsim 1/\Delta t$. On the other hand, the case of replacing missing data with the mean value can be derived from a simpler cost function than that

of the Lomb-Scargle periodogram, where relations between different phase sinusoids of the same frequency are ignored. With the mean first detrended from the data, then the single-frequency fitting and cost functions are given by

$$x(t) \approx x_f(t) = A(f)\, e^{+2\pi\imath f t} \,, \qquad\qquad L(f) = \sum_t w(t)\, |x(t) - x_f(t)|^2 \,, \tag{A.17}$$

with the amplitude solutions and power estimates found to be

$$\hat{A}(f) = \frac{\mathrm{DFT}\{wx\}(f)}{W(0)} \,, \qquad\qquad \hat{P}_{\mathrm{DFT}}(f) = \frac{|\mathrm{DFT}\{wx\}(f)|^2}{W(0)} \,, \tag{A.18}$$

where again $W(f) = \mathrm{DFT}\{w\}(f)$ and $W(0) = n$. Comparing this result to the Lomb-Scargle periodogram relation that we have derived (A.16), we can see that the mean-imputed DFT periodogram shows up in the LSP, but it is shifted and rescaled by terms that vanish when $W(2f) = 0$. If $w(t) = 1$ for all evenly-sampled t, then $W(f) = 0$ for all canonical $f \neq 0$ and so the LSP reduces to the DFT periodogram. In other words, if there are no missing data, the LSP and DFT are strictly equivalent. Note, however, that the ordinary method of inflating the frequency resolution of the DFT periodogram by padding the data with mean values is also not consistent with the LSP value, as padding the data produces a periodogram equivalent to Eq. (A.18). In short, the LSP gives an improved result both when there are missing data and when the frequency resolution is inflated beyond the natural resolution of the data.

A.2 Expectation value of the periodogram

As noted by Scargle (1982), the DFT and LS periodograms tend to be fairly similar in practice, and so one can view the LSP in Eq. (A.16) as being the DFT periodogram $|\mathrm{DFT}\{wx\}(f)|^2/W(0)$ with small corrections. Here we investigate this dominant term of the Lomb-Scargle periodogram under different sampling regimes. For simplification we will assume that the sampling and movement

processes are independent, $\langle w(t)\, x(t')\rangle = \langle w(t)\rangle\, \langle x(t')\rangle$, and that the movement process is stationary with detrended mean and autocorrelation function $\mathrm{COV}[x(t), x(t')] = \sigma(t-t')$. In this case we can express the expectation value

$$\left\langle \hat{P}_{\mathrm{LSP}}(f) \right\rangle \approx \frac{1}{W(0)} \left\langle |\mathrm{DFT}\{wx\}(f)|^2 \right\rangle , \tag{A.19}$$

$$\approx \frac{1}{W(0)} \sum_{tt'} e^{-2\pi\imath f(t-t')} \, \langle w(t)\, w(t')\rangle \, \sigma(t-t') \, . \tag{A.20}$$

For the next step we perform an inverse Fourier transform from the frequency f domain back to the time-lag τ domain to obtain the equivalent relation

$$\mathrm{DFT}^{-1}\Big\{\Big\langle \hat{P}_{\mathrm{LSP}} \Big\rangle\Big\}(\tau) \approx \frac{1}{W(0)} \sum_{ftt'} e^{+2\pi\imath f(\tau-[t-t'])} \, \langle w(t)\, w(t')\rangle \, \sigma(t-t') \, . \tag{A.21}$$

If the data vectors $x(t)$, $w(t)$ are padded with zeros to twice their recorded length $2N$ (on a uniform grid), then the frequency sums evaluate to Kronecker delta functions with the identity

$$\delta_K(t) = \frac{\sum_f e^{+2\pi\imath f t}}{\sum_f} \tag{A.22}$$

and so we have

$$\mathrm{DFT}^{-1}\Big\{\Big\langle \hat{P}_{\mathrm{LSP}} \Big\rangle\Big\}(\tau) \approx \frac{2N}{W(0)} \sum_{tt'} \delta_{\mathrm{K}}(\tau - [t-t']) \, \langle w(t)\, w(t')\rangle \, \sigma(t-t') \, , \tag{A.23}$$

$$\approx \frac{2N}{n(0)} n(\tau)\, \sigma(\tau) \, , \tag{A.24}$$

using the property that the Kronecker delta function is equal to one when its argument is zero and zero otherwise, and where $n(\tau)$ is the number of data pairs recorded with time-lag τ between them. If the data vectors were not padded with zeros to at least twice their recorded length $2N$, and were left with length N, then the second half of the estimate would be a repeat of the first half due to

the periodicity of this representation of the Kronecker delta function.

The pair number $n(\tau)$ is also proportional to the inverse transform of the periodogram of the sampling process $w(t)$. Therefore, back in the frequency domain, the periodogram of the movement process actually represents a convolution of the autocorrelation functions of the sampling process and the movement process. One might imagine de-biasing this estimator by dividing the result by a factor of $n(\tau)$ in the time domain. However, the resulting correlogram would no longer be positive definite, which renders the corresponding periodogram illegitimate in approximating the strictly positive eigenvalue spectrum of a positive-definite covariance matrix.

A.3 Autocorrelation in the sampling schedule

Equation (A.24) shows the direct link between the autocorrelation function $\sigma(\tau)$ and the estimated periodogram $\hat{P}(f)$, as given by the number of observation pairs $n(\tau)$ with time lag τ between them. Importantly, the periodogram is always biased by the sampling schedule through $n(\tau)$. When there are no missing data, $n(\tau)$ is a simple function that decreases linearly with the time lag τ, and its influence on the periodogram is both predictable and mild. If there are missing data with no particular autocorrelation structure in timing of their gaps (uncorrelated and uniformly distributed), then again $n(\tau)$ has a simple structure that does not contaminate the periodogram in a non-trivial way. However, if the sampling schedule is itself periodic, or otherwise temporally autocorrelated, then both the DFT and LS periodograms will exhibit strong biases that say more about the autocorrelation structure of the sampling schedule than that of the signal. This is why the diagnostic argument of `ctmm`'s `plot.periodogram` method will plot both the periodogram of the data and the periodogram of the sampling schedule. Viewing both periodograms side-by-side allows users to check for any possible autocorrelation structure in the sampling schedule that might have propagated into the periodogram of the data.

A.4 Sampling properties and periodogram averaging

The sampling regime intuitively constrains the resolution and bandwidth of the periodogram—i.e., the precision and range of frequencies over which the period can be estimated. Although, the Lomb-Scargle periodogram can be calculated for any frequency, it only contains novel information over some limited set of frequencies. In the case of evenly sampled data where the LSP reduces to the DFT periodogram, this relationship is exact and is summarized by table A.1. In short, temporal resolution translates into frequency range, via the Nyquist frequency $F = 1/(2dt)$, while temporal range translates into frequency resolution. With the canonical set of frequencies described in table A.1, the periodogram contains approximately two locations worth of information per positive frequency. By default, the `ctmm` `periodogram` function inflates this resolution by a factor of two with the `res=1` argument, to make this relationship approximately one-to-one. Inflating the frequency resolution further will cause the estimates $\hat{P}(f)$ to be increasingly correlated between frequencies and lead to a locally smooth periodogram. On the other hand, if the periodogram is evaluated at frequencies beyond the Nyquist frequency, then the periodogram simply repeats itself, as there is no information in the data beyond this cutoff. The Lomb-Scargle periodogram approximately follows the same general relations as the DFT periodogram, though some frequencies can be better sampled than others.

Autocorrelation estimates of any kind can be very noisy for an individual and pooling the estimates of multiple individuals is a good way of reducing this variability. An assumption required to average periodograms is that the sampling frequencies are roughly the same, as differently structured sampling schedules lead to different natural biases of the periodogram. To average multiple individual periodograms, we choose the best frequency resolution (largest T), worst Nyquist frequency (largest dt), and then weight the estimates by their corresponding amount of data. More specifically, we distribute the n degrees of freedom in a dataset over what would be the natural set of frequencies $0 < f \leq F$ for that dataset, regardless of how we fix those parameters for the population

estimate. The worst Nyquist frequency is chosen because evaluating any one periodogram beyond its Nyquist frequency yields a nonsense estimate contaminated by Nyquist frequency periodicities, while the best frequency resolution is chosen because inflating a low-resolution periodogram will only induce correlated errors and we account for this in the average via weighting. This selection criteria is automated by the `ctmm` `periodogram` function when feeding it a list of telemetry objects.

A.5 How periodicity combines with random motion

In App. D we explore periodograms for all of the basic continuous-time movement models, including Brownian motion and Ornstein–Uhlenbeck motion, without any periodicity. These basic movement models provide what a signal analyst might refer to as nuisance "background noise" in which the periodic "signal" exists, in that, for a Gaussian stochastic process the expectation value of the periodogram decomposes into

$$\langle \hat{P}(f) \rangle = \langle \hat{P}_{\mathrm{stochasic}}(f) \rangle + \langle \hat{P}_{\mathrm{deterministic}}(f) \rangle \, , \tag{A.25}$$

where the stochasic component describes an (on average) smooth curve (e.g., $1/f^2$ for Brownian motion) and the deterministic mean component gives us the "peak" or "spike" atop this curve. Therefore the importance of the height of any periodicity is relative to the background curve of a model such as Brownian motion.

A.6 Effect of telemetry error on the periodogram

For additive telemetry errors that are uncorrelated with the movement process, the expectation value of the periodogram of the noisy, observed process $\hat{P}_{\mathrm{data}}(f)$ is given by the sum

$$\langle \hat{P}_{\mathrm{data}}(f) \rangle = \langle \hat{P}_{\mathrm{move}}(f) \rangle + \langle \hat{P}_{\mathrm{error}}(f) \rangle \, , \tag{A.26}$$

of the animal's movement process and the error process. The autocorrelation function of an uncorrelated error process $\sigma_{\text{error}}(\tau)$ is furthermore proportional to a Dirac delta function $\delta(\tau)$, and so from Eq. (A.20) the quantity $\langle \hat{P}_{\text{error}}(f) \rangle$ is approximately constant for all frequencies f. Therefore, the effect of telemetry error is to shift the resulting periodogram vertically along the y-axis and induce further variability in the estimate.

A.7 A gridding algorithm

To apply the periodogram, we want to define a regular temporal grid that is evenly spaced and can accommodate all data points. As GPS fixes can be delayed, there can be variability in the realized sampling intervals of tracking data. To construct a well behaved grid that avoids an unnecessary temporal resolution, we optimize the alignment of our temporal grid relative to the data. Given the data, a regular temporal grid (sampling schedule) can be defined by two parameters: an initial time t_0 and a grid spacing Δt. For the grid spacing Δt, by default, in `ctmm` we use the median realized sampling interval, which performs well if there is a single intended sampling rate. For the initial time, we minimize the cost function

$$\text{COST}(t_0) = \sum_{i=1}^{n} \left| \sin\left(\pi \frac{t_i - t_0}{\Delta t} \right) \right|^2 , \tag{A.27}$$

which is zero if all recorded times are aligned with the grid and greater than zero otherwise. This cost function has the necessary features of being periodic in the parameter t_0 (with period Δt) and increasing monotonically with increasing misalignment. Moreover, this cost function is both analytic and exactly solvable. Differentiating and expanding our cost function, we then have the optimal grid relation

$$0 = \langle S \rangle \cos\left(2\pi \frac{\hat{t}_0}{\Delta t} \right) + \langle C \rangle \sin\left(2\pi \frac{\hat{t}_0}{\Delta t} \right) , \tag{A.28}$$

in terms of the averages

$$\langle S\rangle = \frac{1}{n}\sum_{i=1}^{n}\sin\left(2\pi\frac{t_i}{\Delta t}\right), \qquad \langle C\rangle = \frac{1}{n}\sum_{i=1}^{n}\cos\left(2\pi\frac{t_i}{\Delta t}\right). \tag{A.29}$$

It then derives from Eq.(A.28) the following formula for the optimal initial time

$$\hat{t}_0 = -\frac{\Delta t}{2\pi}\tan^{-1}\left(\frac{\langle S\rangle}{\langle C\rangle}\right). \tag{A.30}$$

This formula is implemented in `ctmm` to propose a default time grid upon which to compute the LSP.

References

Press, W. H., and G. B. Rybicki. 1989. Fast algorithm for spectral analysis of unevenly sampled data. Astrophysical Journal 338:277–280.

Scargle, J. D. 1982. Studies in astronomical time series analysis. II - Statistical aspects of spectral analysis of unevenly spaced data. The Astrophysical Journal 263:835–853.
