# Supplementary material for: Uncovering periodic patterns of space use in animal tracking data with periodograms, including a new algorithm for the Lomb-Scargle periodogram and improved randomization tests
Source: Mov Ecol. 2016 Aug 1;4:19. doi: 10.1186/s40462-016-0084-7 (PMC4968009; doi:10.1186/s40462-016-0084-7)

Appendix to “Periodograms: a non-parametric method to uncover periodic patterns of space use in animal tracking data” by Guillaume Péron, Chris H. Fleming, Rogerio C. de Paula, and Justin M. Calabrese

Appendix C

Simulation study

By comparing outputs from seven scenarios (scenario A to F) we wanted to 1) illustrate how periodograms uncover periodic behaviors even in the presence of large background noise, 2) illustrate the phenomenon of harmonics series occurring in non-sinusoidal or non-elliptical periodic signals, 3) illustrate how artefactual periodicities can be created by improper sampling schedule, and 4) compare variograms and periodograms.

Methods:

In scenario A, we simulated an animal that would repeat the exact same elliptical trajectory every day, and from which a GPS fix would be recorded every two hours. In scenario B, the same animal’s trajectory was affected by random noise under an Ornstein-Uhlenbeck model with foraging (OUF; Fleming et al. 2014a), with autocorrelation parameters $\tau_1 = 10\ \mathrm{hours}$, $\tau_2 = 100\ \mathrm{hours}$ and variance parameter $\sigma = 25\ \mathrm{meters}$. These parameter values were chosen so that the raw data would visually appear uniformly distributed within the home range despite the underlying periodic behavior. In scenario C, a weekly periodicity was added: each week, the last day was spent around the den instead of following the normal route. This signal of weekly periodicity was strongly non-sinusoidal and thereby expected to create a harmonic series. Lastly, in scenarios D and E, the elliptical trajectory was replaced by square- and star-shaped trajectories respectively, to illustrate the creation of harmonic series. In scenario F, we started from scenario A above, but discarded 10 hours of data every 100 hours, effectively creating a periodic sampling schedule that was expected to create an artefactual periodicity in the track record.

Results:

C.1) Uncovering periodic behavior even in the presence of large background noise

Even with a background noise that masked the periodic signal in a static examination of the raw data on a map, the periodicity was retrieved by the LS-periodogram (Fig. C1: Scenarios A & B).

Fig. C1: (a) and (b): Scenario A. An animal follows a strictly elliptical trajectory repeated every 24 hours with a data point every 2 hours. (a) Tracking record. (b) Periodogram. (c) and (d): Scenario B. An animal follows a noisy elliptical trajectory repeated every 24 hours with a data point every 2 hours. (c) Tracking record. (d) Periodogram. (e) and (f): Scenario C. Same as scenario B but each Sunday the animal stays close to its den. (e) Tracking record. (f) Periodogram. Black square symbols: den's location. Vertical lines in periodograms indicate the period of the repeated pattern of space use.

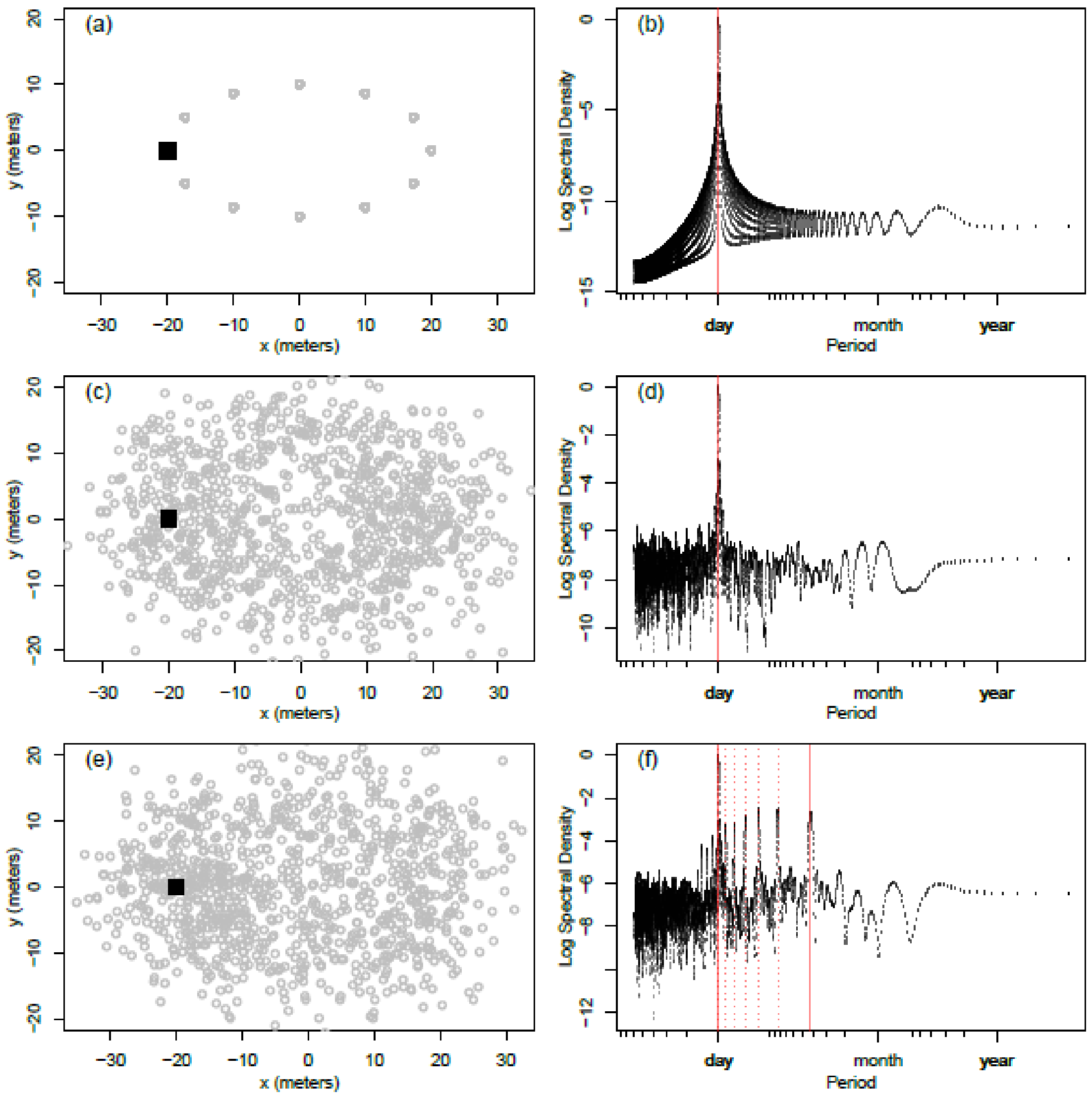

C.2) Interpreting harmonics

Harmonics with periods of a fraction of the main signal occurred when the animal's trajectory was not elliptical (Fig. C2 below: Scenarios D & E) and when the way in which space use was repeated did not follow a regular sinusoid (Fig. C1 in previous page: Scenario C).

Fig. C2: The creation of harmonics when the daily-repeated trajectory is not elliptical. Left column: simulated tracking record. Right column: Lomb-Scargle periodogram. See main text for the description of the simulations. Upper row: Scenario A. Middle row: Scenario D. Lower row: Scenario E.

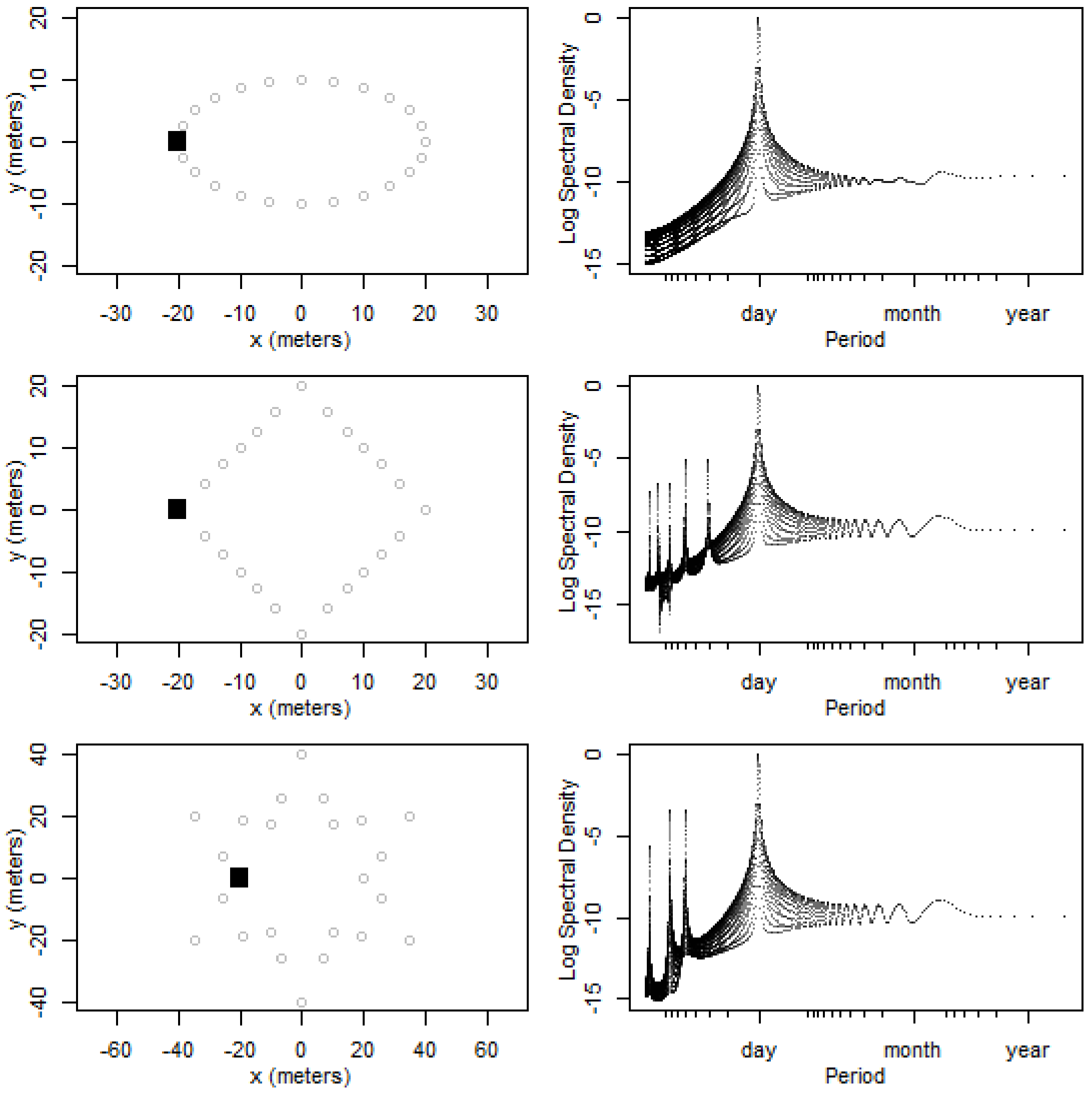

C.3) Identifying artefactual periodicity

The regular gap in the sampling schedule created a strongly bi-periodic signal (Fig. C3): one peak corresponded to the real periodicity in the data, the other corresponded to the periodicity in the sampling schedule. Contrasting the periodogram of the sampling schedule and the periodogram of the simulated data (after re-scaling them for ease of visual comparison) made it possible to correctly assign one of the two periods to the sampling schedule (Fig. C3).

Fig. C3: Artefactual periodicity. Left column: periodogram of the simulated data. Right column: Periodogram of the simulated sampling schedule (see main text). Upper row: Scenario A. No missing data, the periodogram of the data exhibits a single peak corresponding to true periodicity ad the sampling schedule is aperiodic. Lower row: Scenario F. The last 10 hours of each 100 hour period are missing. Both the periodogram of the data and the periodogram of the sampling schedule exhibit a 100 hour periodicity plus associated harmonics of 50 hours, 25 hours, etc. (vertical bars).

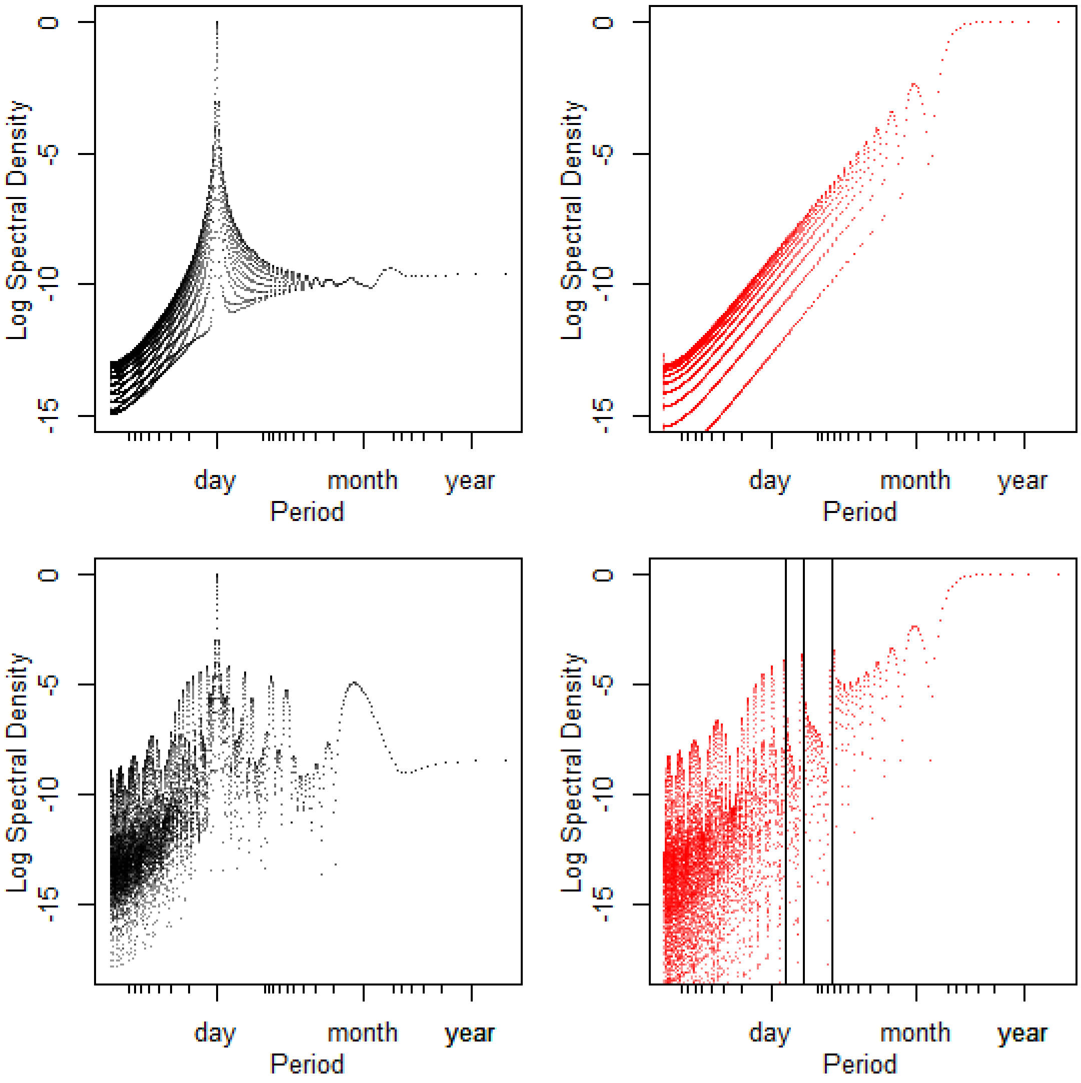

C.4) Comparing variograms and periodograms

In the figure below we compare the variogram and the periodogram in a simulated dataset (from Scenario B described above) and in the albatross study case (see main text). While the periodicity is clearly visible in both the periodogram and variogram, the determination of the period value is clearly more accurate in the periodogram because it is less autocorrelated (less smooth).

Fig. C4: Link between the variogram and periodogram. Left column: In a simulated example (Scenario B described above). Right column: In waved albatross number 3275-30662. Upper panel: Tracking record. Middle panel: Lomb-Scargle periodogram showing the daily periodicity in the simulation and the monthly periodicity in the albatross. Lower panel: Semi-variogram (grey area is the 95% confidence interval). The semivariance decreases when the animal returns back to its initial location. The difference in the width of the peaks is caused by the frequency resolution near the peak, which comes from the sampling interval, and is much coarser in the albatross case than the simulated case because the former has a period of one month which is much larger than the sampling interval. It is also in part due to the relatively low statistical power (only three iterations of the repeated pattern).

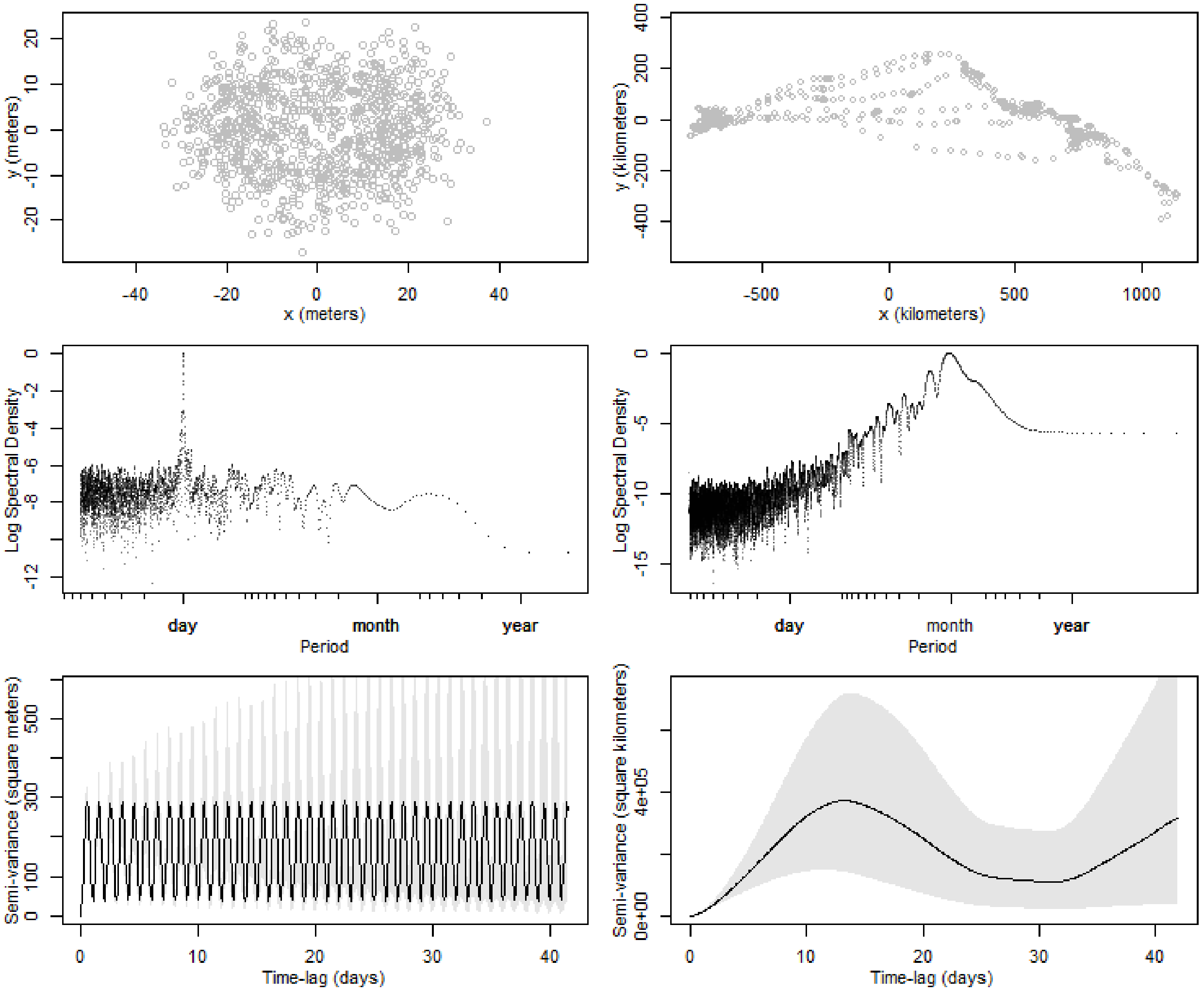

C.5) Periodicity in activity level is different from periodic pattern of space use

We analyzed buffalo Cilla track record for periodicity in her speed, that is the length of each recorded step divided by the duration of the corresponding time interval. We applied the Lomb-Scargle periodogram to this time series of movement speed. The black periodogram corresponds to the movement speed time series (showing a peak below the red arrow), the red periodogram is that of the sampling schedule (showing no peak). The peak, despite its modest size, was highly significant when tested using the null model approach described in the main text (P-value <0.01).

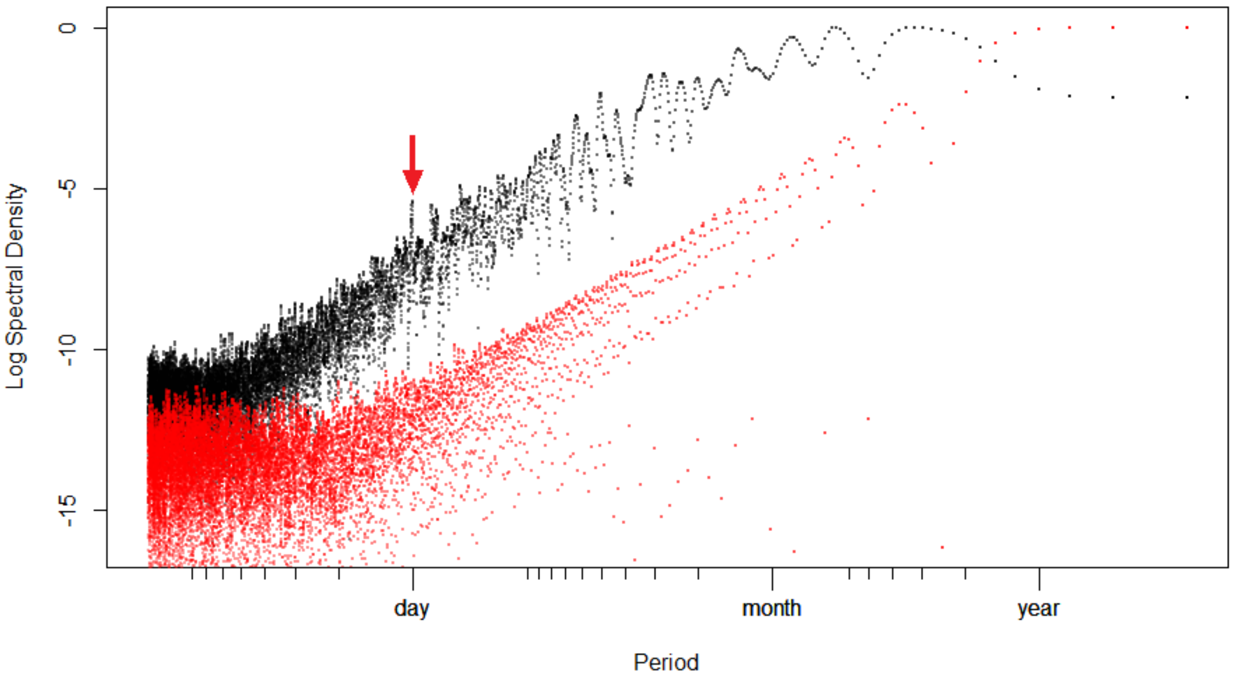

Supplement: Additional file 2: — Simulation study. (PDF 581 kb) [file 40462_2016_84_MOESM2_ESM.pdf]
